# Oxidative stress products and managements in atopic dermatitis

**DOI:** 10.3389/fmed.2025.1538194

**Published:** 2025-05-09

**Authors:** Yingqiang Luo, Jun Hu, Zihao Zhou, Yan Zhang, Yaguang Wu, Jiaying Sun

**Affiliations:** ^1^School of Pharmacy and Bioengineering, Chongqing University of Technology, Chongqing, China; ^2^Department of Neurology, Southwest Hospital, Third Military Medical University (Army Medical University), Chongqing, China; ^3^Department of Dermatology, Southwest Hospital, Third Military Medical University (Army Medical University), Chongqing, China

**Keywords:** atopic dermatitis, skin barrier, oxidative stress, oxidation products, oxidative stress marker, antioxidant therapy

## Abstract

Atopic dermatitis (AD) is a chronic inflammatory skin disorder that affects a significant portion of the global population, severely impacting the quality of life and causing physical and psychological distress of patients. Oxidative stress, resulting from an imbalance between oxidation and antioxidation activities, plays a pivotal role in the pathogenesis of AD. Monitoring oxidative stress products can offer valuable insights into the development of AD and highlight essential clinical and therapeutic effects. Additionally, evidence suggests that antioxidant strategies can alleviate or avert oxidative damage induced by free radicals and offer significant promise in the treatment of AD. In addition to directly utilizing natural products and nanomaterials for antioxidant interventions, these can also be incorporated into hydrogels, which help repair the skin barrier and support the sustained release of therapeutic agents. Furthermore, microneedles provide a minimally invasive method for delivering antioxidants to the deeper layers of the skin, enhancing treatment efficacy. This review aims to summarize the role of the oxidative stress in the pathogenesis of AD, focusing in the main oxidative products (DNA, protein, and lipid oxidation products), as well as antioxidant therapeutic approaches involving natural products, nanomaterials, hydrogels, and microneedles. Understanding these biomarkers and antioxidant therapy approaches provides important insights into the management of AD.

## Graphical Abstract

Generation of oxidative stress products and antioxidant therapy in atopic dermatitis (AD). External stimuli trigger the production of reactive oxygen species (ROS), leading to oxidative stress and the oxidation of DNA, proteins, and lipids. This process subsequently induces skin inflammation, the release of proinflammatory cytokines, and disruption of the skin barrier. Antioxidant therapies involving natural products, nanomaterials, hydrogels, microneedles, and antioxidant enzymes help alleviate these effects and improve the condition of AD. 
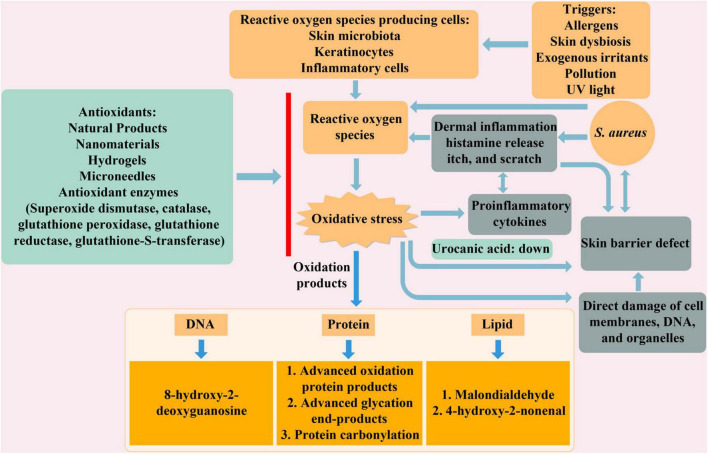

## 1 Introduction

Atopic dermatitis (AD), also recognized as atopic eczema (AE), presents as a chronic, recurrent, and inflammatory skin disorder (1). Affecting approximately 20% of children and up to 10% of adults worldwide (2), AD often manifests at birth or within the first years of life (3), with the majority of cases presenting before the age of two. In the past four decades, the global prevalence of AD has sharply risen, especially in developed countries (4). Predominant symptoms include skin dryness and intense pruritus, often accompanied by erythema, rash, which significantly impact mental health and quality of life (5). Otherwise, AD is associated with various comorbidities, such as food allergies (6), asthma (7), and allergic rhinitis (8).

The pathogenesis of AD is complex and not completely understood. Its multifactorial origins include genetic predisposition (9), environmental factors (10), *Staphylococcus aureus* colonization (11), and neurogenic inflammation (12), all contributing to the AD development. Deficiencies in epidermal barrier function and immune imbalance are intricately linked, with mutations in structural epidermal barrier proteins and immune regulatory factors playing pivotal roles in the pathogenesis of AD (13). Reduced expression of filaggrin is often associated with *S. aureus* colonization. *S. aureus* aggravates AD skin lesions (14) and induces pruritus and skin damage through the V8-PAR1 axis (15). One study has reported that filaggrin mutations are linked to infantile eczema and AD. Individuals carrying filaggrin gene mutations are not only at a higher risk of developing skin dryness on the trunk and extensor surfaces of the limbs in infants aged 3–6 months, but they also face an increased risk of developing eczema and AD (16). During the onset of AD, particularly in the acute phase, changes in the skin barrier activate a Th2-mediated immune response. Chronic skin inflammation in AD patients is caused by persistent Th2 inflammation and skin barrier disruption, accompanied by significant reactive oxygen species (ROS) production (17).

Reactive oxygen species are generated in response to different triggers such as allergens, cutaneous dysbiosis, exogenous irritants, pollutants and UV light (18). In particular, ROS are primarily derived from activated immune cells, including neutrophils and macrophages, as well as keratinocytes under oxidative stress conditions. The accumulation of ROS disrupts the balance between ROS generation and the antioxidant defense mechanisms. Eventually, the excessive oxidative stress is related to the progression of AD (19, 20). In the pathogenesis of AD, ROS derived from activated keratinocytes act in an autocrine and paracrine manner; being important mediators in the main functions of the keratinocyte: maintenance of the skin barrier function, interaction with the skin microbiome and triggering the immune response, including the recruitment of inflammatory cells from the dermis. However, elevated levels of ROS cause excessive oxidative stress and damage cellular components. Additionally, excessive ROS leads to oxidative damage to DNA and proteins, as well as lipid peroxidation of cell membranes, ultimately resulting in cell death (21). Research has indicated that biomarkers linked to oxidative stress, such as urinary 8-hydroxy-2′-deoxyguanosine (8-OHdG), malondialdehyde (MDA), nitrite (NO_2_^–^), nitrate (NO_3_^–^), and biopyrrin, are significantly increased in AD patients (22–24). Furthermore, oxidative stress and inflammation mutually reinforce each other (25), with inflammatory cells producing ROS exacerbating oxidative stress, while ROS and oxidative stress products promote inflammatory responses.

To mitigate or avert oxidative damage induced by free radicals, the body has developed an intricate antioxidant defense mechanism. The skin, in particular, has its own antioxidant defense system that eliminates excess ROS through both enzymatic and non-enzymatic pathways, thereby maintaining redox balance and prevent the damage of ROS to cellular tissues (26). Supplementation with external antioxidants is a crucial strategy to combat oxidative stress, especially in AD patients, enhancing their ability to manage oxidative stress (27). The use of exogenous antioxidants is thus a significant component of antioxidant therapy for AD. Currently, various antioxidant methods have been formulated for the management of AD, including the use of natural product like pterostilbene (28), nanomaterial such as HC-HT-CSNPs (29), SINH-liposome-hydrogel (30), and lipid microparticles loaded with quercetin on microneedles (31), among other antioxidants.

Although ROS contribute to oxidative stress and inflammation, they are also essential for normal physiological functions. Besides direct microbial killing, ROS are also involved in immune responses and emerging as central signaling molecules in the inflammatory response (32). Excessive reduction of ROS may impair these fundamental physiological functions, potentially weakening host defense mechanisms and disrupting normal skin repair processes. Additionally, it introduces the role of antioxidants in the treatment of AD from four therapeutic approaches: natural products, nanomaterials, hydrogels, and microneedles. This review primarily analyzes oxidative stress products in AD at three levels: DNA, protein, and lipid. Additionally, it introduces the role of antioxidants in the treatment of AD from four perspectives: natural products, nanomaterials, hydrogels, and microneedles. Our study aims to explore oxidative stress markers and antioxidant treatment strategies in AD, thereby providing additional therapeutic opportunities for AD management in the future.

## 2 Role of oxidative stress in AD

Atopic dermatitis is a chronic inflammatory skin disease that commonly occurs in children (33). It is characterized by immune activation, epidermal hyperplasia, and defects in barrier function, reflecting potential changes in keratinocyte differentiation (34). The relationship between skin barrier changes and AD has been confirmed in the pathogenesis of the disease (35). Keratinocytes are key contributors to skin barrier function, playing a central role in the formation of the lipid bilayer and the production of filaggrin. Filaggrin is subsequently degraded into urocanic acid, an essential component of natural moisturizing factors that helps maintain skin hydration. Due to the reaction of reactive substances produced in keratinocytes to the environment and endogenous pro-oxidants, the skin has become the main target of response to oxidative stress. As the largest organ and a vital barrier separating the body’s internal environment from the external milieu, the skin is constantly exposed to a variety of external substances, leading to the generation of oxidative and inflammatory mediators (36). Uncontrolled production of ROS and cytokines results in oxidative stress and inflammation. While ROS production is a natural response to environmental changes, prolonged exposure to elevated ROS levels or oxidative stress facilitates the occurrence and exacerbation of skin diseases (37).

Oxidants encompass free radicals or any species containing unpaired electrons, including ROS. Oxidative stress assumes a critical role in AD and other dermatological conditions, evidenced by increased oxidative stress marker levels and diminished antioxidant levels in affected individuals (38). The pathogenesis of AD involves heightened ROS production, as evidenced by elevated ROS levels in skin biopsy specimens from AD patients, assessed using chemiluminescence techniques (39). The colonization of *S. aureus* frequently observed on the skin of AD patients, is associated with the generation of ROS through bacterial enzymes binding to the aryl hydrocarbon receptor (AHR) (40). AHR contributes to skin homeostasis by upregulating barrier-related proteins, including filaggrin (FLG), loricrin (LOR), and involucrin (IVL). However, the protective effects of AHR activation must be balanced against the antagonistic IL-13/IL-4–JAK–STAT6/STAT3 signaling pathway, which disrupts barrier integrity and promotes oxidative stress in AD. Moreover, ROS-induced high-mobility-group-protein B1 (HMGB1) secretion from keratinocytes facilitates *S. aureus* colonization and persistence by disrupting skin barrier integrity through the downregulation of epidermal barrier genes (41). Protein and lipid peroxidation products generated by oxidative stress in keratinocytes contribute to skin barrier dysfunction and exacerbate the progression of AD (42). Inflammatory responses and ROS production are closely intertwined (43). Inflammatory responses enhance the production of ROS, which further amplify inflammation in turn.

Beyond keratinocytes, oxidative stress also influences various immune cells. Dendritic cells exposed to ROS undergo activation, leading to the secretion of pro-inflammatory cytokines that amplify the immune response in AD (44). In the acute phase of AD, Th2-mediated immune reactions initiate the release of pro-inflammatory cytokines, further perpetuating the inflammatory cascade (45). Additionally, keratinocyte-derived cytokines can also activate Th2-mediated responses under oxidative stress conditions, contributing to skin inflammation in AD (46). In addition, oxidative stress promotes the polarization of macrophages toward a pro-inflammatory M1 phenotype, contributing to sustained inflammation (47). Oxidative stress disrupts the balance between regulatory T cells and effector T cells, leading to immune dysregulation and sustained inflammation in AD (48). Oxidative stress also plays a critical role in modulating cellular signaling events in multiple cell types involved in AD pathogenesis. ROS influence signaling pathways such as NF-κB, JAK-STAT, and MAPK, which are involved in keratinocyte function, immune cell activation, and cytokine production (49). The activation of these pathways by oxidative stress exacerbates inflammation and skin barrier dysfunction, further promoting AD progression.

## 3 Marker of oxidative stress in AD

Oxidative stress affects DNA, proteins, and lipids, leading to the formation of various oxidative products in AD. The major oxidative stress markers in AD are summarized in Table 1.

**TABLE 1:** Oxidative stress markers in atopic dermatitis.

Study population	Type	Oxidative stress markers	References
Children, adult	DNA oxidation	8-hydroxy-2′-deoxyguanosine (8-OHdG)	(22, 50, 51)
Children, adult	Protein oxidation	Advanced oxidation protein products (AOPPs) Advanced glycation end- products (AGEs)	(52)
Children, adult			(53, 54)
Children, adult		Protein carbonylation (PC)	(55, 56)
Children, adult	Lipid peroxidation	Malondialdehyde (MDA) 4-hydroxy-2-nonenal (4-HNE)	(57- 59)
Children, adult			(52, 60)
Infant, children	Others	Nitric oxide (NO), nitrite and nitrate Thiol/disulfide balance Biopyrrin	(61, 62)
Infant, children			(63, 64)
Children, adult			(24, 65, 66)

### 3.1 DNA oxidation products-8-hydroxy-2’-deoxyguanosine

**FIGURE 1:**
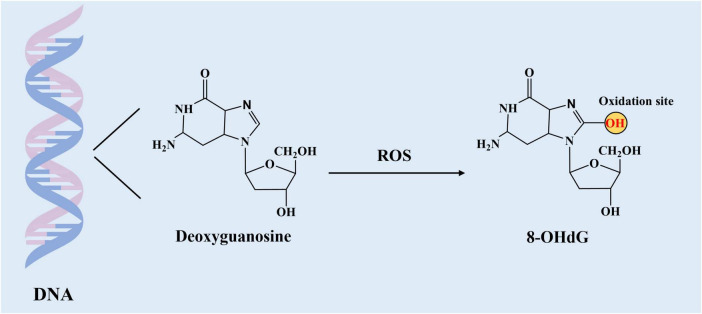
Generation of DNA oxidation products. The C-8 position of guanine in DNA is susceptible to reactive oxygen species (ROS) attack and hydroxylation, generating the adduct 8-hydroxy-2′- deoxyguanosine (8-OHdG).

Oxidative stress-induced DNA damage can be assessed using nucleoside derivatives, which act as indicators of oxidative damage. One of the biomarkers is 8-hydroxy-2′-deoxyguanosine (8-OHdG), generated by the oxidation of the deoxyguanosine at the C-8 position (Figure 1). This biomarker is characterized with sensitive, stable and holistic biomarker of oxidative stress *in vivo* (67, 68). 8-OHdG, a product of damaged DNA is released into the bloodstream as a result of the action of the repair enzyme DNA glycosylase, and it is subsequently eliminated in urine (69).

Detecting levels of 8-OHdG in urine can serve as a means to evaluate oxidative damage to DNA in the evaluation of AD (50, 70). Studies comparing 8-OHdG levels in the urine of AD patients and healthy controls have demonstrated positive correlations between 8-OHdG levels and dermatitis scores, indicating disease severity. Additionally, urinary 8-OHdG levels are markedly elevated in children with AD compared to controls, further supporting its utility as a biomarker for AD. A large-scale study involving 200 children diagnosed with AD found that urinary levels of 8-OHdG were considerably increased in the AD group relative to healthy controls (*p* < 0.001) (22). Children with chronic AD exhibited urinary 8-OHdG levels that were 1.6 times higher compared to in healthy controls, with a trend toward decreasing levels as the patients began to heal (51). However, these observations may not be exclusive to acute exacerbations of AD but could instead reflect general changes seen in inflammatory or infectious disorders (65). Moreover, investigations into psoriasis, another chronic refractory skin disease, have also detected 8-OHdG in the urine of affected individuals (71). The results showed that DNA oxidative damage also existed in psoriasis, and 8-OHdG could also be used to monitor the incidence of psoriasis, with expression levels comparable to those observed in AD.

### 3.2 Protein oxidation products

#### 3.2.1 Advanced oxidation protein products

**FIGURE 2:**
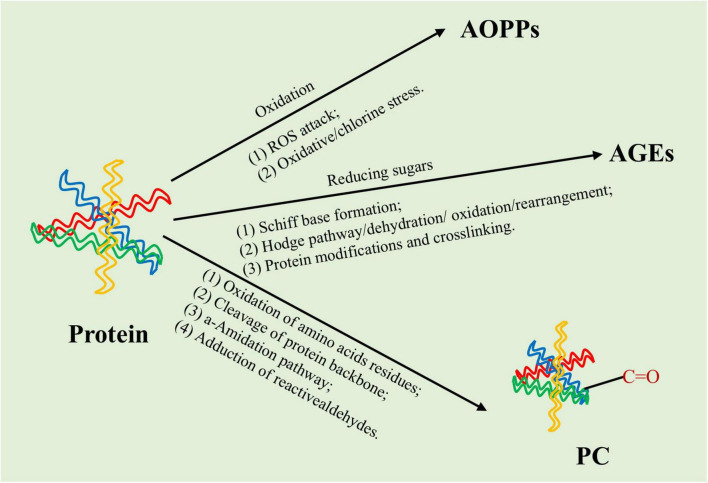
Generation of protein oxidation products. The principal mechanism driving advanced oxidation protein products (AOPPs) generation involves the activated myeloperoxidase-H_2_O_2_-chloride system in neutrophils. AGFs are primarily generated through Maillard reaction, which is roughly divided into initial stage, intermediate stage and final stage. Protein carbonylation (PC) may occur due to direct oxidation of amino acid residues by reactive oxygen species (ROS) or non-oxidative reaction with carbonyl containing oxidized lipids.

Advanced oxidation protein products (AOPPs) constitute a class of complex protein compounds composed of dimethyltyrosine, pentosidine, and carbonyl residues. AOPPs originate from oxidative stress reactions involving plasma proteins and chlorinated oxidants. The principal mechanism driving AOPPs generation involves the activated myeloperoxidase-H_2_O_2_-chloride system in neutrophils, with myeloperoxidase serving as the sole enzyme capable of generating chlorinated oxidants (Figure 2) (72, 73). Hypochlorous acid (HOCl) produced by this system is indicative of AOPPs production. In hemodialysis patients, elevated levels of AOPPs have exhibited a positive correlation with plasma myeloperoxidase activity (74) and oxidized fibrinogen was identified as a principal molecule contributing to the positive chemical reaction to AOPPs (75, 76). In 1996, the first detection of this biomarker of oxidative stress was proposed for plasma in patients with chronic uremia (77). Compared to healthy individuals, AOPPs levels in patients with advanced chronic kidney failure who had not yet undergone dialysis were nearly threefold higher.

As markers of oxygen-mediated protein damage, AOPPs have been identified as indicators of oxidative protein damage and proinflammatory mediators (78, 79). Elevated AOPPs levels have been associated with the advancement of various human diseases and their associated complications, thus serving as markers of oxidative stress across diverse pathologies. Monitoring AOPPs can help predict the development of diseases associated with oxidative stress. In addition to significantly elevated plasma AOPPs levels observed in patients with chronic uremia, similar findings have been demonstrated in other conditions such as cutaneous burns (80). In both groups of second and third degree thermal burns, AOPPs levels were reduced following treatment as a result of decreased levels of oxidative stress. Notably, ROS may contribute to oxidized protein damage within the stratum corneum, thereby disrupting barrier function and exacerbating AD (55). Currently, studies have shown that compared to healthy individuals, patients with AD and chronic urticaria exhibit elevated levels of AOPPs (52). Moreover, an association between AOPPs levels and age has been documented in patients with AD, underscoring the potential utility of AOPPs as biomarkers for disease severity and progression (52).

#### 3.2.2 Advanced glycation end- products

Advanced glycation end-products (AGEs) and AOPPs share structural similarities and exert comparable biological effects. Their accumulation in biological systems results in analogous clinical outcomes. In the context of protein oxidation, the relationship between AGEs and the generation of AOPPs is noteworthy (75). Both AOPPs and AGEs possess similar structures that induce comparable biological effects, and their accumulation in biological systems leads to similar clinical consequences. AGEs, a diverse group of biologically active compounds, were initially identified by French researchers. They are produced through a non-enzymatic glycation process called the Maillard reaction, which uses the carbonyl groups of reducing sugars and the free amino groups of proteins as substrates (Figure 2) (81). AGEs are categorized into endogenous and exogenous sources (82). Endogenous AGEs are generated during normal physiological processes and aging, while exogenous AGEs primarily originate from dietary sources, with their content varying across different foods.

AGEs are produced in a slow and controlled process, accumulating in the body, including the skin (83). The skin is particularly reactive to changes in AGE levels. Their accumulation increases free radical production, stimulates the release of pro-inflammatory factors, and exacerbates inflammatory reactions. AGEs disrupt the dynamic balance of the skin (84), alter the normal substance composition and structure of different skin layers, impair the skin barrier function, and trigger skin issues. The skin barrier function is essential in the onset and progression of AD. Research has documented that AGEs levels are elevated in the keratinocytes of AD patients compared to healthy individuals, with severe AD patients exhibiting significantly higher levels than those with mild AD. However, no significant difference in serum AGE levels was found between typical AD patients and healthy controls (53). Pentosidine, a specific AGE, is closely associated with oxidative stress, with accelerated production observed in oxidative stress-related diseases (85). Pentosidine can serve as a marker for detecting AD (54). Urine tests in AD patients have shown significantly higher levels of pentosidine compared to the healthy controls. Pentosidine levels were significantly higher in AD patients in the acute phase, but reduced during the recovery process, mirroring trends observed for another AD biomarker, 8-OHdG. The consumption of exogenous AGEs increases the risk of developing AD (86), with pregnant women consuming high-AGEs foods potentially exposing their fetus to a higher AGEs environment, thereby increasing the likelihood of AD development.

#### 3.2.3 Protein carbonylation

In addition to AOPPs and AGEs, protein carbonylation (PC) serves as another marker of protein oxidation in patients with AD. From a medical perspective, protein carbonylation has been emphasized as markers of protein oxidation, oxidative stress, and disease progression (52). The oxidative modification of proteins, characterized by the formation and/or introduction of carbonyl groups into proteins, represents a primary indicator of oxidative damage to proteins (87–89). PC, an irreversible oxidative modification (88), is classified into two types based on the origin of the carbonylated product (Figure 2). Consequently, changes in protein conformation following modification typically result in the loss of protein function (90). Proteins undergoing carbonylation exhibit diverse biological significance within biological systems, leading to varied biological effects (88, 91–93). PC is reflected in various diseases, including brain diseases, inflammatory diseases, autoimmune diseases, and aging (94–96), showing important connections between carbonyls in oxidized proteins, oxidative stress, and disease.

Oxidative stress plays an important role in the pathogenesis of AD, with studies demonstrating elevated levels of PC in dry skin and AD skin lesions (56). Levels of PC in the skin of AD patients are elevated and positively correlated with the severity of the disease (55). Sampling from AD patients utilizing the tape stripping method has revealed increased levels of PC in the stratum corneum, a phenomenon similarly observed in patients with psoriasis (97). The accumulation of PC in the skin contributes to transepidermal water loss and altered dermal matrix accumulation (98). ROS are implicated in inducing damage to cuticular oxidation proteins, disrupting skin barrier function, and exacerbating the development of AD.

### 3.3 Lipid peroxidation products

#### 3.3.1 Malondialdehyde

**FIGURE 3:**
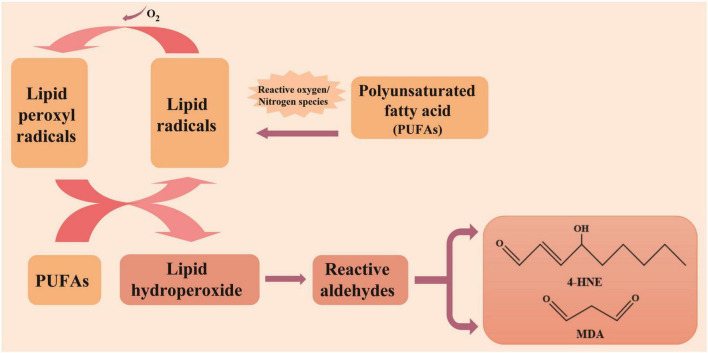
Generation of lipid oxidation products. 4-hydroxy-2-nonenal (4-HNE) and Malondialdehyde (MDA) are prevalent lipid oxidation products in atopic dermatitis (AD). Lipid oxidation is closely related to polyunsaturated fatty acids (PUFAs), typically progresses through three stages: initiation, propagation, and termination.

Lipids are the most impacted biomolecules in oxidative stress-induced damage, and the identification of these end-products in inflammatory diseases suggest that lipid peroxidation is crucial in such diseases (99). Malondialdehyde (MDA), an important metabolite of arachidonic acid and unsaturated fatty acids possessing multiple unsaturated C-C double bonds in lipids, was the main and most widely studied compound (100) derived from lipid peroxidation following oxidative damage from oxygen radical attack (Figure 3) (101, 102). Upon exposure to oxidative stress, excessive accumulation of ROS disrupts the structure and function of the cell membrane, alters its permeability, and causes lipid peroxidation, inducing the production of MDA.

Malondialdehyde is produced *in vivo* through both enzymatic and non-enzymatic oxidation, making it a widely monitored biomarker for oxidative stress across various diseases and particularly favored for characterizing oxidative stress in AD patients (57). A likely correlation exists between serum antioxidant levels and MDA in individuals with eczema (58), with an inverse correlation between antioxidant levels and MDA levels and decreased serum levels of antioxidant vitamins in patients than in healthy persons. In children with AD, serum levels of MDA were found to be on average 0.055 units higher, while melatonin (a hormone with antioxidant activity) levels were approximately 3.05 units higher compared to controls. The increase in serum melatonin in AD might represent a compensatory mechanism to reduce skin inflammation by attempting to alleviate excessive oxidant production (59). For the antioxidant properties of quercetin, liposomes incorporating quercetin gel have demonstrated both protective and therapeutic effects on skin eczema (103). Treatment with quercetin-loaded liposomes resulted in significantly reduced skin pathological symptoms compared to untreated counterparts, accompanied by reduced levels of MDA in both liver and skin tissues.

#### 3.3.2 4-hydroxy-2-nonenal

4-hydroxy-2-nonenal (4-HNE) is a lipid peroxide produced by polyunsaturated fatty acids (PUFAs) in response to oxidative stress (Figure 3). It belongs to the same class of lipid peroxidation products as MDA. Both 4-HNE and MDA are extensively studied markers of lipid peroxidation, with MDA recognized as a mutagenic product of lipid peroxidation and 4-HNE recognized as the most toxic product of lipid oxidation (104), worsening the damage resulting from oxidative stress (105, 106). 4-HNE is classified as an α, β-unsaturated aldehyde and possesses three functional groups: carbon-carbon double bonds, carbonyl groups and hydroxyl groups. Due to the existence of the conjugated system of C = C bonds and carbonyl groups, 4-HNE can provide partial positive charge to the carbon at position three and become efficient electrophiles, allowing it to react with essential biological molecules, including proteins and DNA (107–109). 4-HNE can also be generated through both enzymatic and non-enzymatic reactions from the breakdown of ω-6 PUFAs. However, the chemical reaction mechanism of 4-HNE formation remains unclear. Several mechanisms have been proposed over the years. The earliest suggestion was that 4-HNE was formed from PUFAs catalyzed by transition metal ions (102, 110). The formation mechanism of 4-HNE is attributed to the observation of hydroperoxides as an intermediate product.

As a secondary messenger of free radicals and growth regulators, 4-HNE participates in various pathophysiological processes and act as a bioactive marker, especially in oxidative stress related to diseases (111). Skin homeostasis plays an important role in the development of AD. Studies have found that cigarette smoke can trigger the generation of ROS (112). Exposure to cigarette smoke can affect skin homeostasis due to oxidative and inflammatory reactions, as well as inducing lipid peroxidation, leading to increased levels of 4-HNE (113). In a study examining oxidative stress markers in exhaled breath condensates of children with AD, 4-HNE levels were elevated in AD patients but did not differ significantly from those in healthy children (60). Another study found a similar phenomenon by measuring serum 4-HNE levels in AD patients, with 4-HNE concentrations comparable to those in healthy subjects.

### 3.4 Others

#### 3.4.1 Nitric oxide, nitrite and nitrate

Nitric oxide (NO), a free radical gas containing unpaired electrons, is the smallest biologically active molecule produced by mammalian cells. It exhibits lipophilicity and possesses a high degree of diffusivity in tissues and cells (114). NO is primarily synthesized in organisms by nitric oxide synthase (NOS), an enzyme divided into three subtypes: inducible iNOS, endothelial eNOS, and neurogenic nNOS (115). Additionally, NO can be produced via non-canonical pathways, either through the reduction of nitrite to NO or by the sequential conversion of nitrate to nitrite and then to NO (116). With diverse biological functions, NO is essential for regulating the body in both healthy and disease states (117). In dermatology, NO is implicated in mechanisms underlying inflammatory or immune-mediated dermatoses, skin infections, skin cancers, and wound healing (118). Skin inflammation is significantly affected by NO (119). Keratinocytes, fibroblasts, and immune cells synthesize NO, and each contributing to the occurrence of inflammatory responses. The importance of NO as a mediator of skin inflammation is underscored by the delicate balance between its production and degradation, which is closely related to the onset and development of inflammatory skin diseases. Overproduction of NO has been linked to conditions such as AD and psoriasis (120–122).

Direct measurement of nitric oxide (NO) presents challenges due to its extremely short half-life in the bloodstream, typically disappearing within seconds, and its involvement in various biochemical reactions within living organisms (123, 124). NO is highly reactive owing to its unpaired electrons, capable of engaging in oxidative stress by reacting with free radicals, with the primary oxidative metabolites being nitrite and nitrate (125). A study found elevated levels of IgE, nitrite, and nitrate in the plasma of AD patients (61). Furthermore, a study involving 88 cases of AD and 12 cases of non-AD founded that serum nitrate levels not only showed a significant increase in children with AD, but also associated with the severity of the disease (62).

#### 3.4.2 Thiol/disulfide balance

Thiols have emerged as a novel marker of oxidative stress, representing a class of organic compounds containing sulfhydryl groups. These sulfhydryl groups, commonly known as thiols, consist of sulfur and hydrogen atoms bonded to carbon atoms (126). The sulfhydryl groups within thiols provide protection against oxidative stress by scavenging ROS through enzymatic or non-enzymatic mechanisms. Thiols serve as physiological agents for neutralizing free radicals and other ROS (127). Through a series of reactions, thiols undergo modifications and react with oxidants to form disulfide bonds (128). The oxidation of thiols to disulfides is facilitated by through various processes, crucially involving three distinct mechanisms (129). Disulfide compounds formed as a result of these reactions can undergo reversible conversion to a thiol structure, thereby maintaining the dynamic balance of thiol/disulfide equilibrium (130).

Disruption of this balance has associated with the development of certain inflammatory diseases. In infants diagnosed with AD, thiols have been found to be significantly reduced compared to in comparison to healthy controls, while disulfide levels were markedly elevated, indicative of dysregulation in the thiol/disulfide balance favoring peroxidation (63). However, contrasting findings were observed in another study (64), which serum disulfide levels were observed to decrease in AD children in comparison to healthy children, leading to a reduction in the disulfide/natural thiol and disulfide/total thiol ratios.

#### 3.4.3 Biopyrrin

Biopyrrin is a metabolite derived from the oxidation of bilirubin. Bilirubin, acting as a potent ROS scavenger, reacts with ROS, thereby exerting a robust antioxidant effect (131). Upon oxidation, bilirubin is converted into various forms of bilirubin oxidation metabolites (BOMs), comprising at least seven hydrophilic metabolites that are promptly excreted into urine because of their hydrophilic nature (132). These metabolites, including biopyrrin, can be detected by anti-bilirubin monoclonal antibody 24G7 (133, 134). A key advantage of using biopyrrin as an oxidative stress marker is its ability to provide real-time insights into the dynamic changes of oxidative stress through urine detection, thereby reflecting the oxidative stress status associated with various disease states.

Biopyrrin holds promise as a novel biomarker for AD (66). Urinary excretion of biopyrrin was markedly increased in pediatric patients experiencing acute exacerbations of AD, with levels averaging 1.8 times higher than those observed in healthy individuals (65). Bilirubin oxidation was enhanced in the diseased skin of patients with AD and levels of the oxidized metabolite biopyrrin detected in urine correlated with the severity of AD (24). Moreover, urinary biopyrrin levels were positively correlated with serum IgE and TARC/CCL17 expression. Biopyrrin expression was higher in AD lesions compared to normal skin, as detected by the 24G7 antibody. These findings highlight the potential utility of biopyrrin as a valuable biomarker for assessing oxidative stress and the severity of AD.

## 4 Manage oxidative stress in AD

Immunity and inflammation are pivotal in the pathogenesis of AD. Current therapeutic strategies predominantly focus on anti-inflammatory or immune-modulating agents. While the majority of patients respond favorably to topical corticosteroids (135), calcineurin inhibitors (136), and immunosuppressants (137), these treatments are often associated with significant adverse effects during long-term use (138). Oxidative stress is positively correlated with factors influencing the onset and advancement of AD. Moreover, prolonged exposure to oxidative stress impacts the condition of keratinocytes, leading to alterations in skin barrier function and cell death. Therefore, considering the use of antioxidants to mitigate AD is an essential strategy in the management of AD.

### 4.1 Natural products

Plants are the primary source of natural antioxidants (139), with antioxidant compounds primarily synthesized as secondary metabolites. Numerous plants and their derivatives exhibit antioxidant properties and frequently possess other significant biological activities. Due to their low toxicity, these natural antioxidants have been extensively utilized in the prevention and management of diseases related to oxidative stress (140). Plant-derived antioxidant compounds can be categorized into several groups: phenolic acids, phenolic diterpenes, flavonoids, volatile oils, carotenoids, and anthocyanins (141, 142). These compounds are abundant in herbs, spices, seeds, essential oils, fruits, and vegetables (143). Additionally, plants and foods containing vitamins and certain trace minerals contribute to the antioxidant process and constitute essential components of natural antioxidants (144, 145).

#### 4.1.1 Natural extracts

Plants typically contain several highly active antioxidant compounds that can be extracted using various technical methods (146). The antioxidant effects of plant extracts are related to the chemical and physical properties of these compounds and operate through multiple mechanisms (147–149). The therapeutic potential of natural extracts for treating AD has been thoroughly explored through both *in vivo* and *in vitro* studies. A study involving 20 patients with mild to moderate AD demonstrated that a cream containing 100,000 IU of superoxide dismutase (SOD) and 4% plant extracts significantly alleviated AD symptoms and was effective across all phases of the disease (150). The therapeutic efficacy of this cream is attributed to the synergistic actions of SOD and the plant extracts, including antioxidant, anti-inflammatory, and additional beneficial properties. Resveratrol, a naturally occurring polyphenol abundant in grapes and berries, has shown positive therapeutic effects on skin disorders (151), potentially affecting inflammation through its antioxidant activity and free radical scavenging properties (152). Intragastric administration of resveratrol has been shown to ameliorate AD in mice induced by dinitrochlorobenzene (DNCB), by downregulating chemokine and proinflammatory factor levels and upregulating the expression of skin barrier proteins (153). Another study also demonstrated that topical formulations based on the antioxidant properties of resveratrol can reduce ROS, inhibit inflammatory responses, and improve skin barrier function (154). Analysis of the chemical composition of Lentinula edodes ethanolic extract revealed that polyphenols are the main antioxidant components, along with flavonoids, β-carotene, and lycopene. This ethanolic extract has shown to decrease serum IgE levels, downregulate the expression of inflammatory cytokines, and alleviate AD symptoms (155). Additionally, some natural extracts can exert antioxidant and anti-inflammatory effects and regulate the Nrf2/HO-1/NQO1 and NF-κB/MAPK signaling pathways to treat AD (156, 157).

#### 4.1.2 Vitamins

Vitamins are a group of organic compounds crucial for maintaining normal physiological functions in the body and can be categorized into fat-soluble and water-soluble groups (158). Most vitamins are obtained through the daily diet. The primary sources of vitamins A, C, and E are fresh vegetables and fruits, while vitamin D is primarily biosynthesized through the skin under sunlight. These four vitamins inherently possess antioxidant properties, allowing them to function as antioxidants (159, 160).

There is a significant connection between vitamins and skin diseases. Maintaining a reasonable and stable vitamin level is crucial for preserving normal skin health (161). β-carotene (provitamin A) exhibits antioxidant and immunomodulatory effects, enhancing skin barrier function and reducing inflammation levels in hairless mice with oxazolone-induced AD (162). Vitamin C contributes to the formation of skin structure and skin antioxidation (163), ameliorating chronic inflammation and positively impacting AD. In groups supplemented with vitamin E, levels of oxidative stress markers were decreased, and a reduction in vitamin E concentration contributed to the progressing of AD in dogs (164). Another study supports that vitamin E supplementation can lower IgE levels in AD patients and improve AD symptoms (165). Vitamin D supplementation is beneficial for AD treatment and can be used to treat AD in children and dogs (166, 167). Furthermore, these vitamins can work synergistically to enhance AD treatment. For instance, the dermatitis score was lower in the group receiving both vitamin D and vitamin E compared to groups receiving either vitamin alone (168). However, there are differing views on the efficacy of vitamins in improving AD. Diets rich in antioxidant compounds can reduce the risk of AD. Taking in β-carotene and vitamin E is negatively correlated with AD, whereas vitamin C intake does not show a consistent correlation (169). Additionally, higher concentrations of vitamin C in breast milk are linked to a lower risk of atopy in infants, whereas vitamin E shows no consistent relationship with AD (170).

#### 4.1.3 Minerals

Trace minerals, also known as trace elements, play an important role in maintaining overall nutrition and health (171), playing vital roles in the metabolism and physiological processes of the body. Several trace elements are involved in the redox reaction process. Selenium (Se) is an essential trace mineral that forms a key part of selenoproteins, which primarily exert their nutritional functions through a family of 25 selenoproteins. Se is an enzymatic antioxidant that has no antioxidant effect by itself but participates in selenoproteins as redox-active selenoenzymes to safeguard against oxidative damage (172, 173). Inhibiting iron death by regulating selenoprotein GPx4 plays a significant role in improving skin inflammation (174). Keratinocytes, which are integral to the skin barrier function, are also implicated in skin disorders such as AD. The supplementation of Se and selenoprotein SEPP1 can alleviate the oxidative stress and toxicity of 4-ClBQ-induced keratinocytes (175). Nevertheless, a 12 weeks study revealed that selenium-enriched yeast supplementation did not result in significant improvements in AD severity, indicating no substantial difference before and after supplementation (176).

Zinc, another essential nutrient for skin health, is abundantly present in the epidermis (177). Although zinc itself is not an antioxidant and is redox-inert, it contributes to oxidative defense through several mechanisms (178, 179). Decreased zinc levels have been observed in AD patients, taking zinc supplements orally may help those who are zinc deficient to manage AD (180). Zinc is commonly used as a nutritional supplement for cosmetic purposes and in the management of AD. However, caution is warranted regarding zinc concentration, as excessive intake can lead to zinc toxicity (181). The efficacy of zinc supplementation in treating AD remains controversial, with some studies suggesting no significant benefit, while others indicate potential therapeutic effects (182).

### 4.2 Nanomaterials

Nanotechnology has seen extensive development across various disciplines and facilitates the synthesis of nanoparticles via bottom-up and top-down strategies (183). Conventional antioxidants frequently encounter challenges, including limited permeability, poor aqueous solubility, instability, and low bioavailability (184). Consequently, nanomaterials have become a pivotal area of research dedicated to improving the efficacy of antioxidants. The utilization of nanotechnology presents a promising avenue for overcoming the limitations associated with conventional antioxidants, thereby exhibiting significant potential in the realm of antioxidant therapy. Antioxidant nanomaterials can be broadly categorized into two types: those with inherent antioxidant properties and antioxidant delivery nanomaterials. The first type includes nanomaterials that possess antioxidant properties independently, without the need for functionalization with antioxidants. The second type comprises nanomaterials that do not inherently have antioxidant properties but can be used to load and deliver antioxidants, thereby exerting antioxidant effects.

#### 4.2.1 Nanomaterials with intrinsic antioxidant activity

Among antioxidant nanomaterials, several types possess inherent antioxidant properties, most of which are metal nanoparticles. These nanomaterials can mimic the efficacy of antioxidant enzymes like catalase (CAT), superoxide dismutase (SOD), and glutathione peroxidase (GPx). The antioxidant enzyme activity of nanomaterials is influenced by factors including size, morphology, surface modification, and composition (185). Catalase-like nanoenzymes, a category of nanomaterials with intrinsic CAT activity, operate by decomposing H_2_O_2_ into H_2_O and O_2_ (186). Superoxide radicals (O_2∙_^–^), a type of ROS generated during metabolic processes, are converted into H_2_O_2_ and O_2_ by SOD, using metal as a cofactor (187). GPx, the final antioxidant enzyme, catalyzes the reduction of H_2_O_2_ or organic hydrogen peroxide to H_2_O or alcohol in the presence of reduced glutathione (188). Cerium oxide nanoparticles (189) exhibit SOD-like activity, and their PEGylation can enhance the survival rate of keratinocytes while significantly reducing intracellular ROS levels. Cobalt oxide nanoparticles, synthesized via a one-pot method, demonstrate three enzymatic catalytic activities. These nanoparticles can protect keratinocytes from hydrogen peroxide-induced ROS and toxicity, alleviating the symptoms of AD (190). Furthermore, hematoxylin and eosin and toluidine blue staining indicated a decrease in epidermal thickness and a reduction in the number of mast cells in the treated group.

#### 4.2.2 Antioxidant delivery nanomaterials

Most antioxidants have low bioavailability due to their inherent properties (191, 192). However, nanotechnology can enhance the antioxidant effect by preparing nanoparticles as carriers for these antioxidants, allowing for targeted and controlled release (193). Currently, a variety of antioxidant delivery systems have been developed to transport natural and synthetic antioxidants, antioxidant gases, genes, and other antioxidant compounds, thereby significantly expanding the scope of antioxidant delivery nanomaterials (194). Nanoparticles prepared with the natural polyphenol antioxidant hydroxytyrosol, hydrocortisone, and chitosan have shown significant improvement in the pathological characteristics of AD in mice. Compared to the AD group, the treatment group exhibited decreased expression levels of IgE, histamine, PGE2, VEGF-α, and AD-related Th1 and Th2 cytokines. Histological examination also demonstrated that HC-HT-CS-NPs exerted a therapeutic effect on AD (195). Additionally, HC-HT-CS-NPs demonstrated favorable safety in healthy individuals (196).

### 4.3 Hydrogels

Hydrogel is polymer material with a three-dimensional porous structure formed by physical or chemical crosslinking of polymer chains. Hydrogels exhibit notable hydrophilicity, enabling them to absorb water and biological fluids, and they possess excellent moisturizing and air permeability properties (197). Due to their high water content, structural similarity to natural tissues, and favorable biocompatibility, hydrogels are widely used in biomedical fields, particularly for drug delivery, facilitating controlled release and enhancing efficacy (198–200). Additionally, the physical and chemical properties of hydrogels can be easily modified to impart various functions, including antioxidant properties (201). Antioxidant hydrogels can be categorized into self-antioxidant hydrogels and those combined with antioxidant components (202).

These hydrogels have been applied in skin diseases and have shown promise as carriers for the local treatment of AD. Lignin, a polyphenol-containing substance extracted from lignocellulosic biomass, has the capability to scavenge ROS. Hydrogels prepared by crosslinking lignin with polyethylene glycol exhibit CAT and superoxide SOD enzyme-mimicking properties and possess antioxidant capacity (203). These hydrogels can treat AD by reducing skin oxidative stress. They protect HaCaT cells from oxidative stress damage caused by H_2_O_2_. In DNCB-induced AD mice, treatment with these hydrogels reduced dermatitis scores and epidermal thickness, inhibited inflammation, alleviated DNA oxidative damage, and decreased Th2 cytokine levels.

Furthermore, cerium oxide nanoparticles known for their high ROS scavenging ability, have been incorporated into hydrogels. By adjusting pH and crosslinking sodium alginate polymer with Ca^2+^, a CENP-sodium alginate hydrogel was prepared. This hydrogel effectively mimics CAT and SOD activity, protecting cells from oxidative stress damage. In AD mice, treatment with this hydrogel reduced epidermal thickness, decreased 8-OHdG accumulation, lowered Th2 cytokine and IgE levels, and reduced mast cell infiltration, showing its therapeutic potential for AD management (204).

### 4.4 Microneedles

The skin is composed of three primary layers: the epidermis, dermis, and subcutaneous tissue (205). The stratum corneum, an integral part of the skin barrier, is formed through the differentiation of keratinocytes and serves as the primary protective layer against external injury and stimulation (206). However, this physiological structure presents a challenge for transdermal drug delivery, limiting the bioavailability of drugs administered through the skin. Microneedles, ranging in length from 25 to 2,000 μm (207), offer a promising transdermal technology by piercing the stratum corneum and crossing the skin barrier to reach all layers of the skin. This technology has been widely used in the treatment of skin diseases, enabling the delivery of antioxidants into the skin or using the inherent antioxidant properties of the microneedles to address oxidative stress-related skin conditions (208–210).

In the treatment of AD, microneedles loaded with epigallocatechin gallate, a potent antioxidant, and L-ascorbic acid as a stabilizing reductant, prepared from poly-γ-glutamic acid, exhibit multiple beneficial functions. These microneedles improve DNCB-induced AD in mice through antioxidant, anti-inflammatory, and immunomodulatory effects (211). Treatment outcomes include reduced epidermal thickness, decreased mast cell infiltration, and lower levels of serum IgE and histamine. Microneedles incorporating natural polyphenols such as curcumin and gallic acid, prepared from PLGA/HA in a double-layer configuration, enable rapid treatment and long-term management of AD (212). The curcumin and gallic acid -loaded microneedles provide immediate antioxidant and anti-inflammatory effects, alleviating AD symptoms in the short term, while the embedded PLGA needle mediates the sustained release of curcumin for long-term improvement. Post-treatment, AD mice exhibited reduced dermatitis scores and improved pathological conditions, with a significant decrease in ROS levels in the lesional skin after 56 days. Additionally, a novel polydopamine nanozyme integrated with near-infrared-responsive microneedles was developed using natural dopamine. Hyaluronic acid was used for the backing layer, and hyaluronic acid methacrylate was employed for the tip, enabling antioxidant treatment of AD (213). PDA MNs + NIR treatment alleviated AD symptoms and inhibited Th2 immune-related reactions. Measurement of the DNA oxidative stress marker 8-OHdG revealed that the PDA MNs + NIR group significantly downregulated its levels.

## 5 Conclusion

Atopic dermatitis is a chronic inflammatory skin disorder characterized by disruptions in skin barrier integrity and immune dysregulation. The complex pathogenesis of AD involves oxidative stress, which induces several cellular damages in keratinocytes, impairs skin barrier function, and exacerbates the inflammatory response. This oxidative damage results to modifications of DNA, proteins, and lipids, culminating in the formation of various oxidation products. While several antioxidant strategies, such as the use of natural products, nanomaterials, hydrogels, and microneedles have shown promise in mitigating oxidative stress and alleviating AD symptoms, there remain knowledge gaps. Further research is required to fully elucidate the specific oxidative modifications underlying AD pathology, the precise mechanisms of action of antioxidant therapies, and the strategies for optimizing these treatments for clinical application. Addressing these gaps will be essential for developing more effective therapeutic strategies and monitoring tools to improve the management of AD.
